# The Impact of Caregiving Intensity and Religiosity on Spouse Caregivers’ Health and Mortality in the United States (2004–2014)

**DOI:** 10.1093/geroni/igaa057.1658

**Published:** 2020-12-16

**Authors:** Athena Koumoutzis, Nader Mehri

**Affiliations:** 1 Miami University, Oxford, Ohio, United States; 2 Miami University, OXFORD, Ohio, United States

## Abstract

Prior research has indicated that religiosity may buffer against the deleterious effects of caregiving. However, research is lacking in examining the role of religiosity and caregiving intensity in the context of caregiver wellbeing and mortality. Data come from the Health and Retirement Study (2004-2014 waves) and consisted of spousal caregivers and noncaregivers (n= 49,638 person-spells). Pearlin’s Stress Process Model (1990) informed this study to analyze how religiosity impacts caregiver self-rated health and mortality by comparing the intensity of provided care among spousal caregivers and spousal noncaregivers. This study used two indicators to measure religiosity: 1) the importance of religion in life and 2) frequency of attending religious services. Bivariate probit model was used to model the impact of caregiving intensity and religiosity on self-rated health and all-cause mortality. After controlling for sociodemographic and health covariates, results showed that only the importance of religion in life predicted a better self-rated health among high intense spouse caregivers defined by providing >=14 hours of care per week. Findings suggest religiosity may buffer the adverse effect of caregiving stress on health for high intense spousal caregivers. Development and maintenance of religiosity may enhance positive aspects of caregiving and decrease caregiver burden.

